# UbiProt: a database of ubiquitylated proteins

**DOI:** 10.1186/1471-2105-8-126

**Published:** 2007-04-18

**Authors:** Alexander L Chernorudskiy, Alejandro Garcia, Eugene V Eremin, Anastasia S Shorina, Ekaterina V Kondratieva, Murat R Gainullin

**Affiliations:** 1 Nizhny Novgorod State University, 23 Gagarin Ave., Nizhny Novgorod, Russia; 2 Central Research Laboratory, Nizhny Novgorod State Medical Academy, 10/1 Minin Sq., Nizhny Novgorod, Russia; 3 Institute of Applied Physics RAS, 46 Ul'yanov St., Nizhny Novgorod, Russia

## Abstract

**Background:**

Post-translational protein modification with ubiquitin, or ubiquitylation, is one of the hottest topics in a modern biology due to a dramatic impact on diverse metabolic pathways and involvement in pathogenesis of severe human diseases. A great number of eukaryotic proteins was found to be ubiquitylated. However, data about particular ubiquitylated proteins are rather disembodied.

**Description:**

To fill a general need for collecting and systematizing experimental data concerning ubiquitylation we have developed a new resource, UbiProt Database, a knowledgebase of ubiquitylated proteins. The database contains retrievable information about overall characteristics of a particular protein, ubiquitylation features, related ubiquitylation and de-ubiquitylation machinery and literature references reflecting experimental evidence of ubiquitylation. UbiProt is available at  for free.

**Conclusion:**

UbiProt Database is a public resource offering comprehensive information on ubiquitylated proteins. The resource can serve as a general reference source both for researchers in ubiquitin field and those who deal with particular ubiquitylated proteins which are of their interest. Further development of the UbiProt Database is expected to be of common interest for research groups involved in studies of the ubiquitin system.

## Background

Ubiquitin is a small (76 amino acids) protein that has an ability to be linked to other intracellular proteins via a covalent isopeptide bond [1]. Such a posttranslational modification of target proteins named "ubiquitylation" leads to a manifestation of various biological effects of ubiquitin. Ubiquitylation is implicated in protein degradation via an intracellular ATP-dependent proteolytic system [2]. Additionally, it participates in several non-proteolytic events. It is common knowledge that the ubiquitin system mediates throng of essential cellular outcomes, including a cell cycle control, an inflammatory response, carcinogenesis and many others, therefore ubiquitylation is a phenomenon of great importance for cell vital functions [3].

A diversity of known biological effects of ubiquitylation is realized through modification of a huge variety of target proteins followed by alteration of their functions. A rapidly growing number of experimental evidence on protein ubiquitylation determines a general need for such data collecting and systematization. We try to solve this problem by elaborating a new resource, the UbiProt Database, a knowledgebase of ubiquitylated proteins. This project aims to summarize a significant volume of data concerning ubiquitylation and to provide essential information on target proteins.

## Construction and content

UbiProt is developed and deployed with an open source software. A database management system is MySQL 4.0. The software is developed on the basis of PHP 4.0.3 including some additional modules like SMARTY and PEAR. Web interface software uses PHP+SMARTY template framework.

All data included were experimentally obtained by various research groups and can be verified using respective references. The main content was obtained from several large-scale proteomic studies [4-7]. The rest of target proteins were acquired from original research articles containing direct evidence for ubiquitylation of particular proteins. A manual annotation was performed for all database entries.

**Figure 1:**
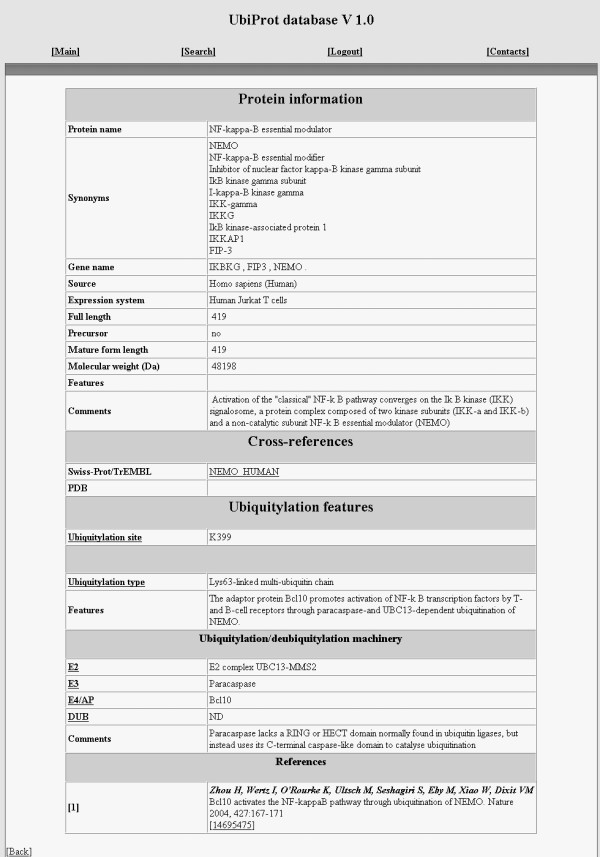
**Structure of a typical UbiProt entry**. This entry contains information about an ubiquitylated NEMO protein.

**Figure 2:**
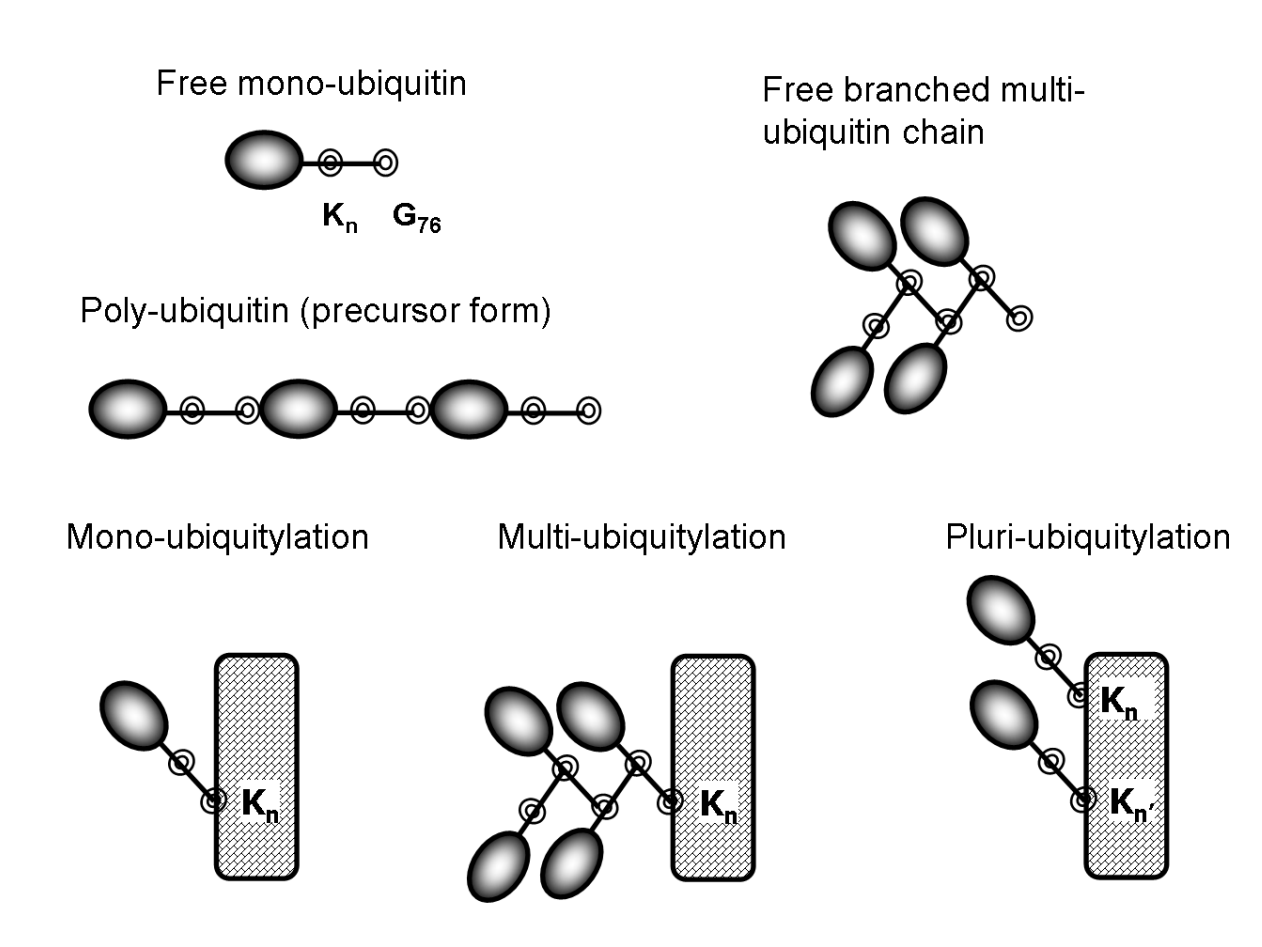
**Different forms of ubiquitin and ubiquitin-modified proteins**. Ubiquitylation may result in addition of a single ubiquitin moiety or a branched multi-ubiquitin chain to the target protein lysine(s). Note that a ubiquitin molecule possesses 7 inner lysine residues that can serve as attachment sites of the next ubiquitin moiety, resulting in the formation of the chains that have a different structure and topology. Functionally significant amino acids are marked as follows: K_n _and K_n' _– lysine residue(s) that can serve as attachment sites of the ubiquitin moiety; G_76 _– ubiquitin C-terminal glycine residue participating in the isopeptide bond formation. Poly-ubiquitin refers to a ubiquitin fusion protein, a precursor form polymerized "head-to-tail".

Each entry possesses a block structure summarized in a single page (Figure 1) to provide highly structured and easy-retrievable information on each ubiquitylated protein. The first information block contains overall characteristics of a particular protein, i.e. a name and synonyms, genes, a source organism and an expression system in case of recombinant proteins, as well as some basic properties such as molecular weight, full length and mature form length. The next block describes protein ubiquitylation features and includes a description of modified lysine residue(s) and an ubiquitylation type: a number of attached ubiquitins (mono-ubiquitylation, multi-ubiquitylation, pluri-ubiquitylation) and a structure of the branched multi-ubiquitin chains. These details are especially important because different kinds of ubiquitylation have a different impact on a fate of the modified protein. For instance, a multi-ubiquitin chain linked by the Lys48-Gly76 isopeptide bond targets a protein for proteasome-mediated degradation, while the Lys63 assembled chains participate in a DNA repair and other non-catabolic processes [8]. On the other hand, mono-ubiquitylation often serves as an endocytic signal for a diverse set of membrane proteins [3]. A diversity of ubiquitin forms and ubiquitin-protein conjugates is illustrated in Figure 2. It also reflects specific terms used in UbiProt in accordance with the terminology offered by H.P. Jennissen [1]. We should also note that there is no universal terminology for ubiquitin system, and ubiquitylation-related terms vary from paper to paper. For example, some authors use the term "poly-ubiquitylation" for a type of modification defined as "multi-ubiquitylation" in Jennissen's terminology.

The following information block provides information on the respective ubiquitylation and deubiquitylation machinery: cognate ubiquitin-conjugating components E2 and E3, deubiquitylating enzyme(s) (DUB), non-enzymatic adaptor proteins (E4/AP), if these components are known.

**Figure 3:**
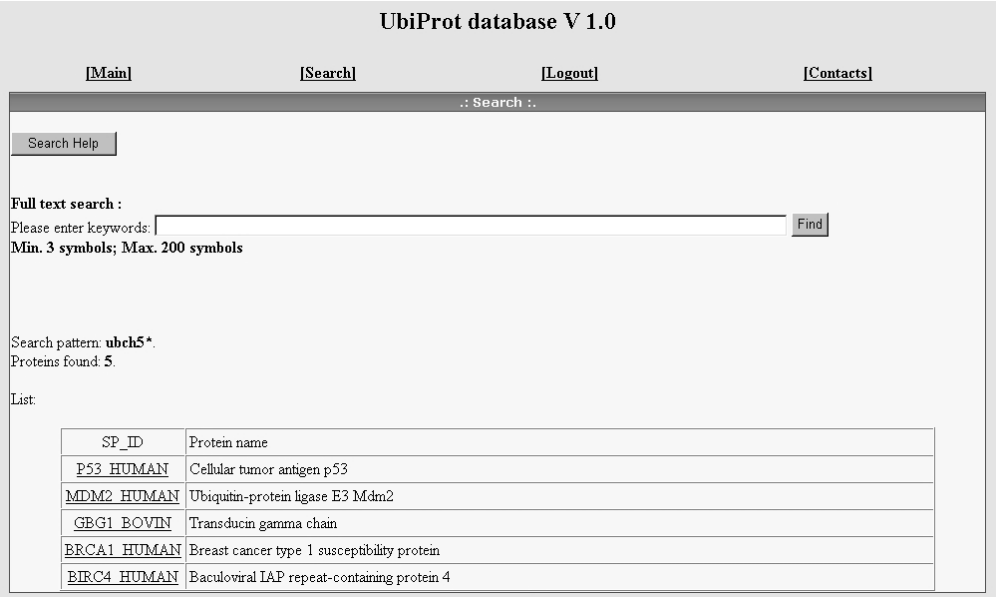
**Example of full-text search results**. The search pattern is "ubch5*" that corresponds to the particular E2 ubiquitin-conjugation enzyme (UbcH5). The result shows proteins to be modified by the specified enzyme.

Each protein entry also contains literature references with the PubMed links [9] denoting primary research articles from which ubiquitylation details have been obtained. Cross-reference to the respective Swiss-Prot/TrEMBL entry is also available [10]. A link to the Protein Data Bank entry is added in case when 3D structure of the protein from the same source is available [11]. The dataset is supplied with a full-text search tool with a possibility of using Boolean operators. An example of search results is given in Figure 3. Additionally, the whole dataset can be viewed using a [View all proteins] option.

## Utility and discussion

UbiProt Database is considered to be a useful tool for different purposes, but mainly for identification of proteins posttranslationally modified by ubiquitin in high-throughput studies. A computational biology (e.g. comparative and evolutionary analysis of protein ubiquitylation) also offers a promising field of a database application. The dataset collected will provide an insight into the ubiquitin-dependent mechanisms controlling essential cellular events and, in future, help to develop new compounds for treating ubiquitin-associated human diseases.

At the present time the information about 400 individual proteins from different organisms is collected and this work is still in progress. Our group permanently analyses a broad array of literature data (approximately 40 journal articles per week) in order to collect new information on the target proteins, identified ubiquitylation sites, structures of multi-ubiquitin chains and features of the ubiquitylation machinery. A continuous renewal of the corresponding fields as well as an insertion of new information blocks, especially concerning a biological function of ubiquitylation, are provided. Our database undergoes updating as soon as appropriate confident information becomes available. We also plan to extend our dataset with a detailed description of up-stream and down-stream components of ubiquitin system, together with comprehensive information on the domain structure of target proteins including ubiquitin-binding domains (reviewed in [12]).

UbiProt is more convenient in terms of search of information about ubiquitylated proteins than more general databases. Although databases like Swiss-Prot and Human Protein Reference Database [13] also contain data about ubiquitylation, there are several problems with a retrieval of the information of interest. First of all, one trying to obtain information about ubiquitylation from Swiss-Prot using the SRS system [14] will face some difficulties with a query formulation, as far as the SRS poorly works with complex queries [15]. A query should be formulated very precisely, so it is necessary to find at least one entry prior to the main search. In addition, even precise queries work well only for proteins with an identified ubiquitylation site(s). An amount of proteins with unknown ubiquitylation sites is much more bigger, and in this case query formulation rules may differ. Another problem is search redundancy. A search for ubiquitylated proteins in Swiss-Prot most likely will return not only pure ubiquitylated proteins, but also numerous enzymes of the ubiquitylation cascade.

Besides several search problems mentioned above, there is lack of data in the established databases. Many known substrates of ubiquitylation do not appear as ubiquitylated proteins in Swiss-Prot (e.g. p53, BRCA1, MDM2, Ymer and others), for many other proteins precise ubiquitylation sites are not designated. Human Protein Reference Database also cannot serve as a complex reference source for ubiquitylation, because it contains data only about 18 ubiquitylated proteins, without details about an ubiquitylation type and respective enzymes. This can be due to the insufficient use of data from the proteomic studies dedicated to ubiquitylation. Only 2 recent papers presenting results of high-throughput analysis [4,5] are reviewed in Swiss-Prot at the moment. Our dataset is based also on another proteomic works published so far [6,7,16].

UbiProt aims to collect data from a number of resources including cited databases to make the information easily accessible after validation and annotation. It is more specific and comprehensive comparing to general sequence databases such as Swiss-Prot.

All scientists working on protein ubiquitylation are encouraged to join collaboration in keeping the database up-to-date by submitting additional information and comments. A downloadable Excel form can be used for submitting new data.

## Conclusion

UbiProt Database is a comprehensive resource on ubiquitylated proteins, aimed to systematize information on protein ubiquitylation and to make it available for further analysis and use. The resource is considered to be useful as a general reference source both for researchers in the ubiquitin field and those who deal with particular ubiquitylated proteins of interest. Its biological utility and application also includes identification of proteins posttranslationally modified by the ubiquitin in high-throughput studies and bio-computational analysis of the ubiquitin-protein conjugates. It may help to understand the mechanisms of ubiquitylation and to develop new compounds for manipulating the ubiquitin-dependent pathways.

Further development of the UbiProt Database is expected to be of common interest for research groups involved in studies of the ubiquitin system.

## Availability and requirements

UbiProt can be accessed on a public Apache powered website at . The website was tested with the most of commonly used Web browsers. It is freely available for non-commercial use. For any questions regarding commercial use please contact the corresponding author.

## Abbreviations

ATP – adenosine triphosphate

Lys – lysine

Gly – glycine

DUB – deubiquitylating enzyme

SRS – Sequence Retrieval System

## Authors' contributions

AC has carried out data analysis, annotation process and linked the dataset with the related protein databases, and drafted the manuscript.

AG has participated in data analysis and coordination of the study and helped to draft the manuscript.

EE has designed the database structure and built the website.

Both AS and EK have participated in the development and maintenance of the database, and carried out data annotation.

MG has conceived of the study, participated in its design and coordination and helped to draft the manuscript.

All the authors have read and approved the final manuscript.
